# Supplementary material for: Multidecadal, continent-level analysis indicates agricultural practices impact wheat aphid loads more than climate change
Source: Commun Biol. 2022 Jul 28;5:761. doi: 10.1038/s42003-022-03731-z (PMC9334390; doi:10.1038/s42003-022-03731-z)

Supplementary Information

Multidecadal, continent-level analysis indicates agricultural practices impact wheat aphid loads more than climate change

Xiao Sun, Yumei Sun, Ling Ma, Zhen Liu, Qiyun Wang, Dingli Wang, Chujun Zhang, Hongwei Yu, Ming Xu, Jianqing Ding*, Evan Siemann

*Correspondence: jding@henu.edu.cn

This PDF file includes:

Supplementary Figures 1 to 6

Supplementary Figure 1. Test of publication bias for the studies of aphid loads vs. time in Figure 2. This funnel plot shows the dependence of study precision (inverse of standard error) on the residuals of the individual paper slopes vs. the contientnal average. Visual inspection and a non-significant Egger test indicate that the plot is symmetrical and there was no publication bias.

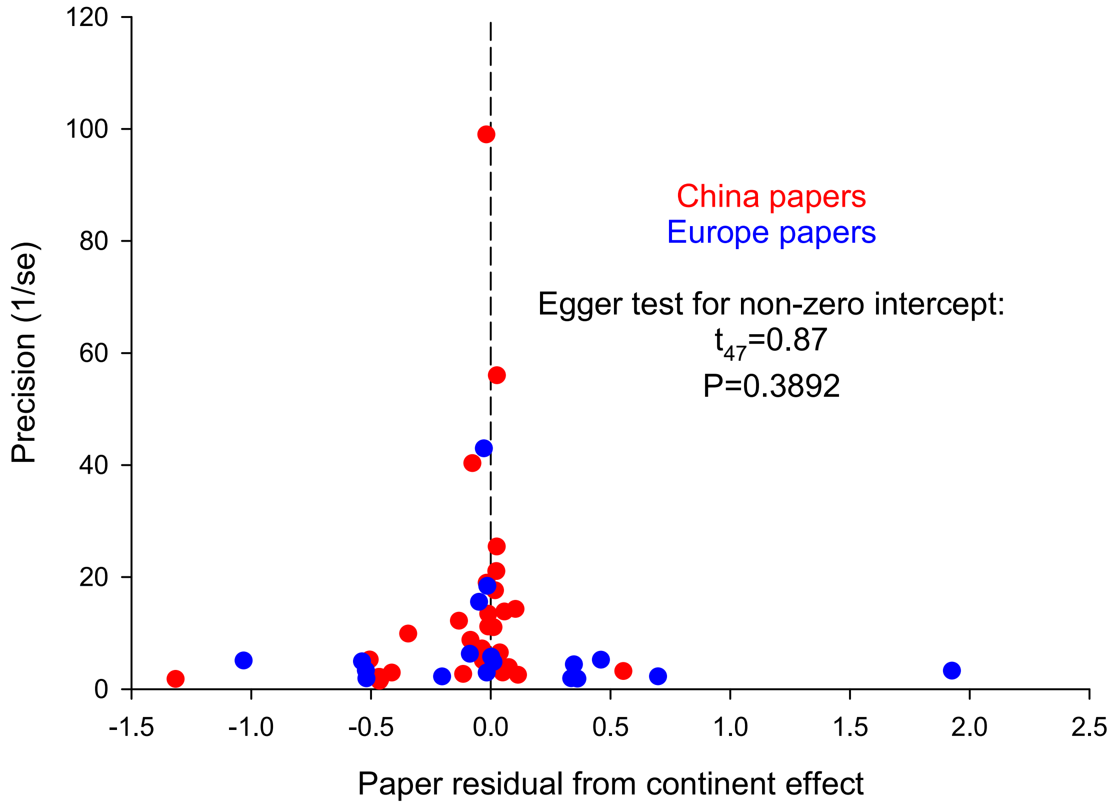

Supplementary Figure 2. Natural enemy abundances in different times of the year in China during 1980-2017. *P* and R^2 values are from regressions. Lines indicate significant linear relationships between natural enemy abundances and time.

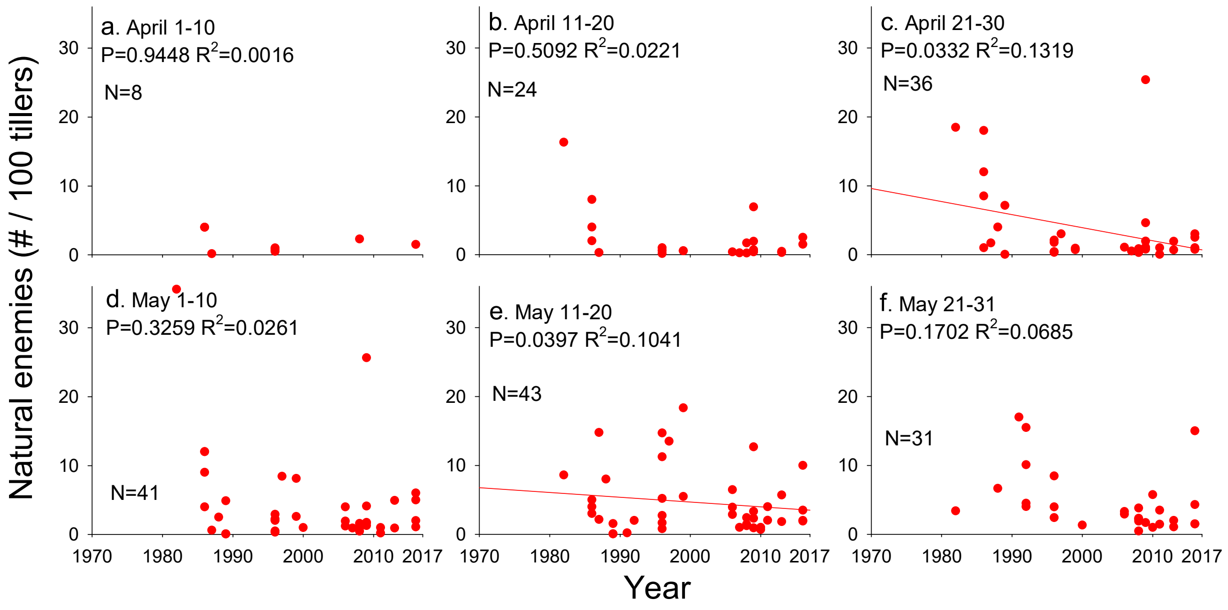

Supplementary Figure 3. Trends of natural enemies in China (a) and Europe (b) from 1980 to 2017. Enemies were reported as lacewings (Neuroptera), ladybirds (Coccinellidae), midges (Cecidomyiidae), hoverflies (Syphidae), spiders (Araneae), unknown parasitoids, unknown predators, or unknown natural enemies.

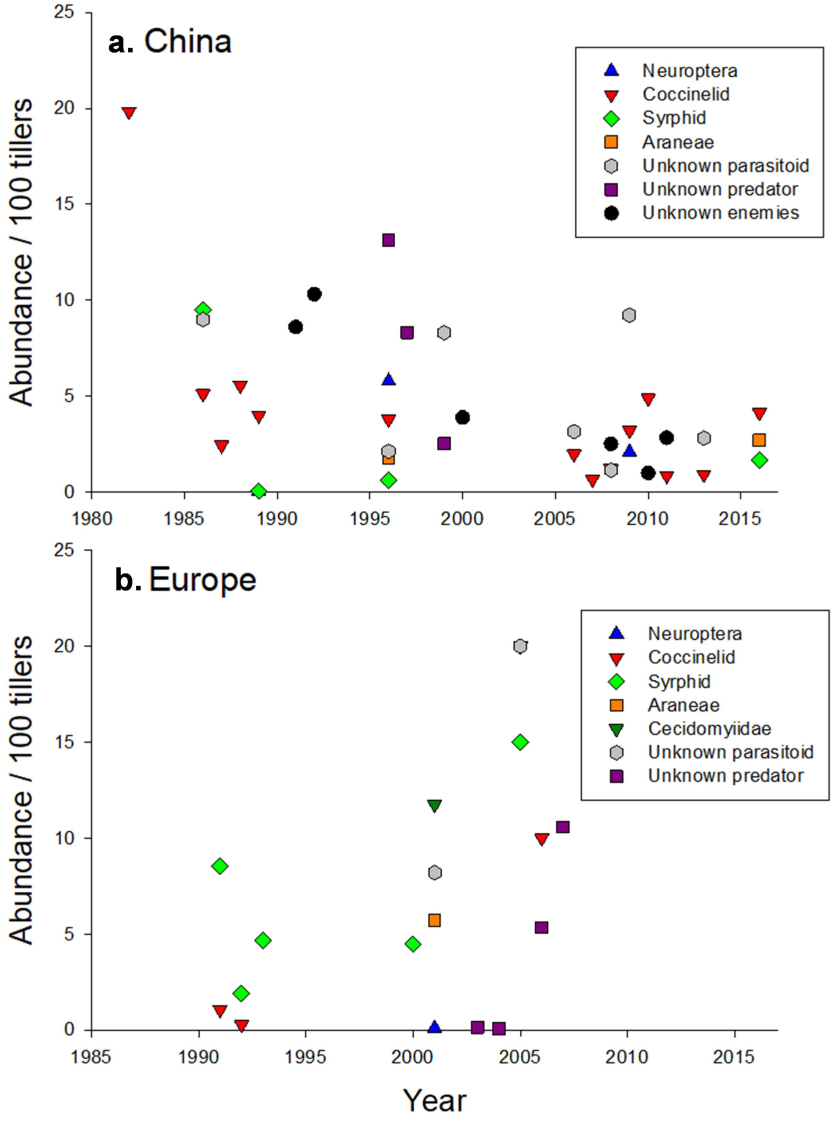

Supplementary Figure 4. Trends in average monthly temperature changes by province in China (accessed at https://www7.ncdc.noaa.gov) and by country in Europe (accessed at https://climateknowledgeportal.worldbank.org/) during 1971-2016. Early is March [China] or May [Europe]. Mid is April [China] or June [Europe]. Late is May [China] or July [Europe]. Winter is December to February [China & Europe]. Station numbers for provinces in China were: Anhui=582030, Gansu=535330, Hebei=546020, Henan=570830, Hubei=574610, Jiangsu=582380, Ningxia=536140, Shandong=548230, Shanxi=537720, Shaanxi=536460, Sichuan=563840, Xinjiang=2241714.

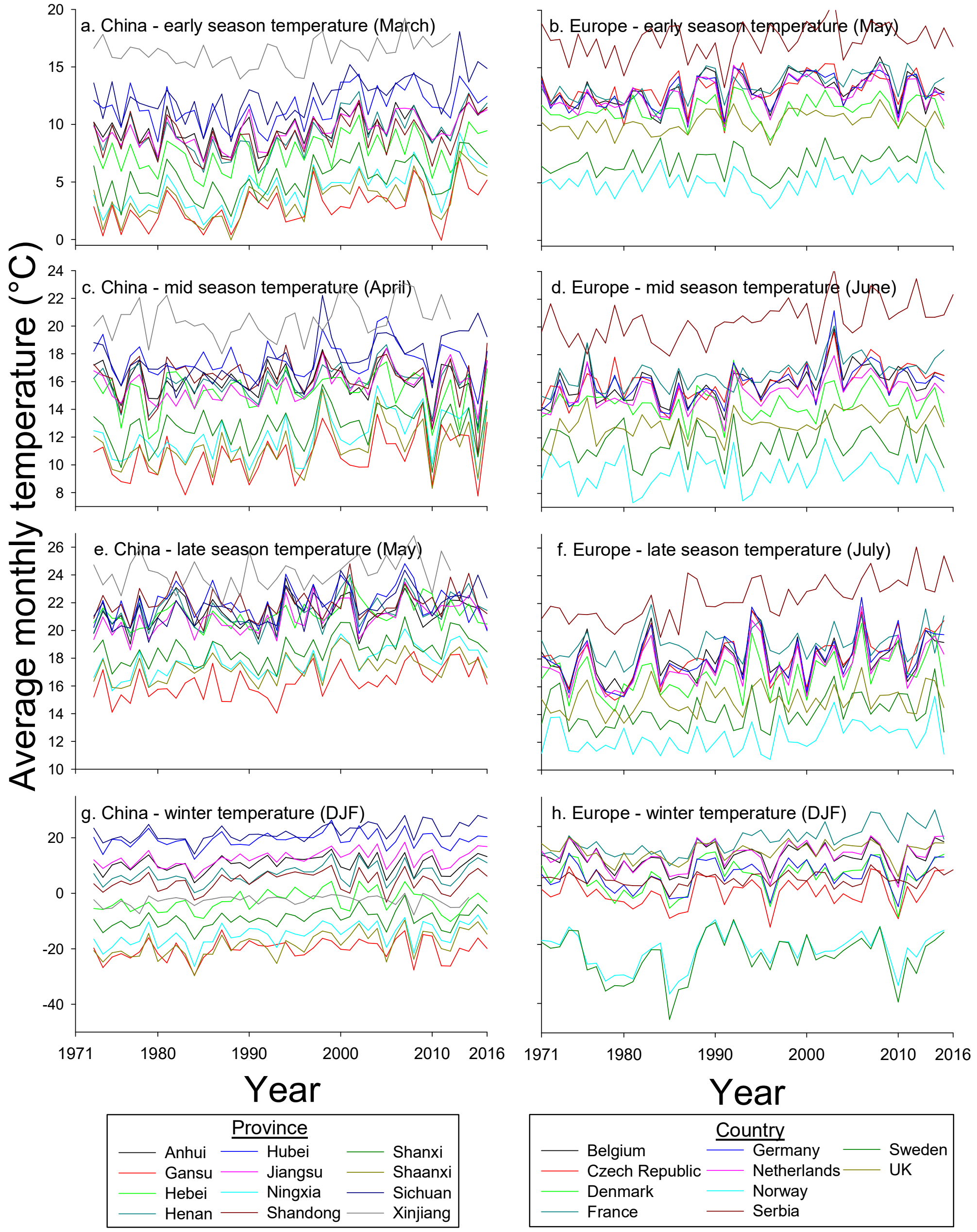

Supplementary Figure 5. Annual monthly average temperatures by province in China and by country in Europe from 1970 to 2016. Early is March [China] or May [Europe]. Mid is April [China] or June [Europe]. Late is May [China] or July [Europe]. Winter is December to February [China & Europe].

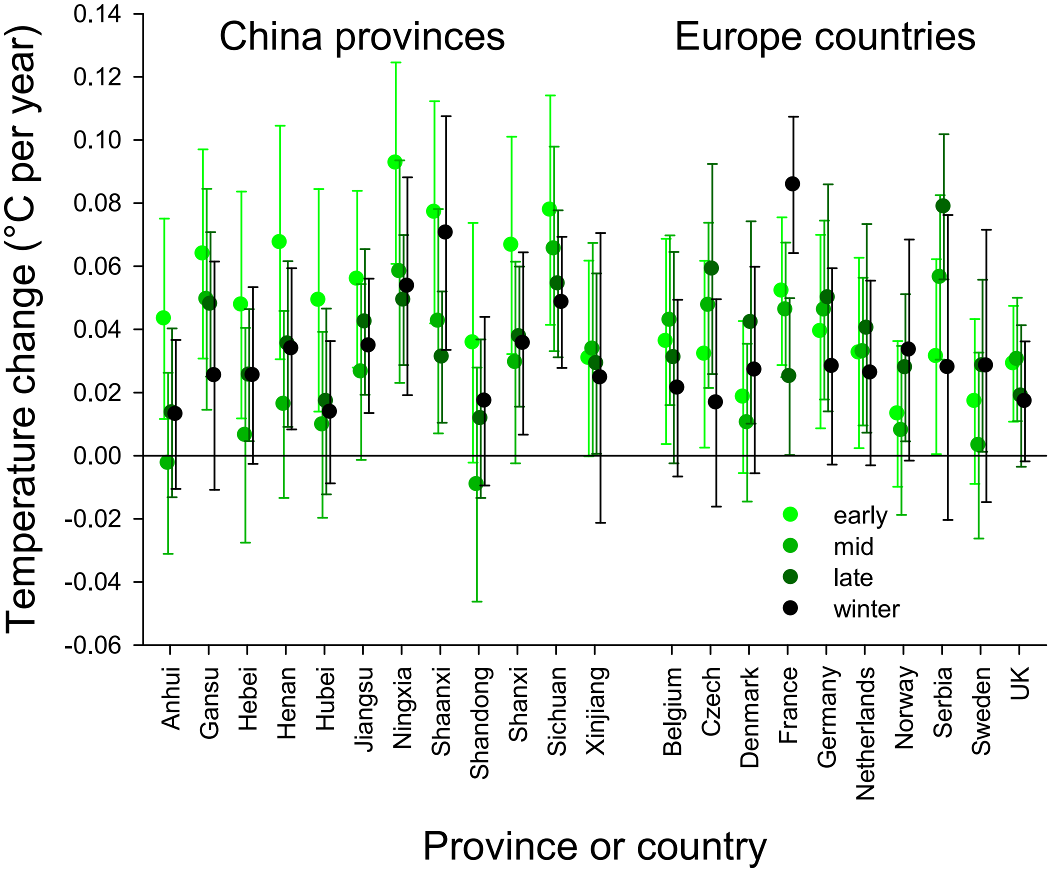

Supplementary Figure 6. Trends of wheat cultivation in Europe (blue) and China (red). Provinces or countries with significant positive patterns of proportion of land in wheat cultivation over time were Anhui, Henan, Denmark, France, Germany, Norway, Sweden and UK and those with significant negative patterns were Gansu, Hebei, Hubei, Hunan, Shaanxi, Shanxi, Shandong, Xinjiang, and Czech Republic.

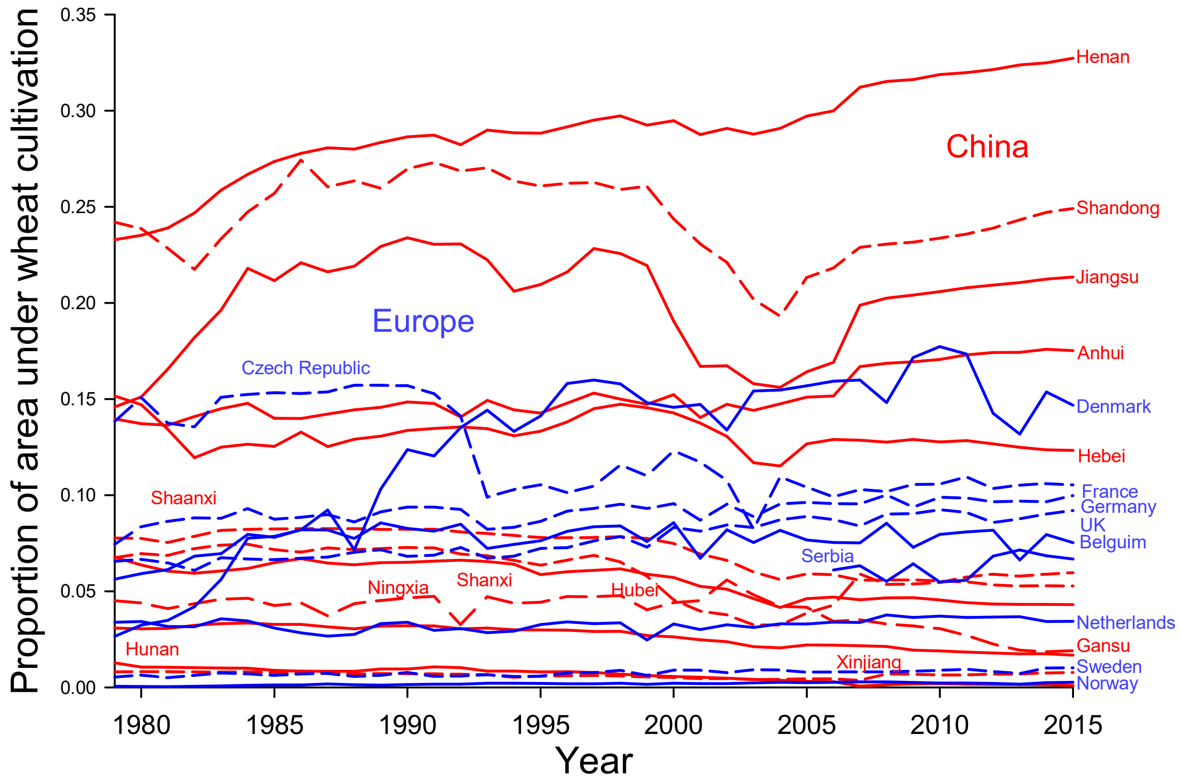

Supplement: Supplementary file 2 — Supplementary Information [file 42003_2022_3731_MOESM2_ESM.pdf]
