# Supplementary material for: Multidecadal, continent-level analysis indicates agricultural practices impact wheat aphid loads more than climate change
Source: Commun Biol. 2022 Jul 28;5:761. doi: 10.1038/s42003-022-03731-z (PMC9334390; doi:10.1038/s42003-022-03731-z)
Supplement: Supplementary file 3 — Description of Additional Supplementary Files [file 42003_2022_3731_MOESM3_ESM.pdf]

Description of Additional Supplementary Files

File name: Supplementary Data 1

Description: The source data behind the graphs in the paper.

File name: Supplementary Data 2

Description: Temperatures (ºC) by province in China and by country in Europe during 1971-2016.

File name: Supplementary Data 3

Description: Data details for land-use intensity.
