# Supplementary material for: Multidecadal, continent-level analysis indicates agricultural practices impact wheat aphid loads more than climate change
Source: Commun Biol. 2022 Jul 28;5:761. doi: 10.1038/s42003-022-03731-z (PMC9334390; doi:10.1038/s42003-022-03731-z)
Supplement: Supplementary file 5 — Supplementary Data 2 [file 42003_2022_3731_MOESM5_ESM.docx]

**Supplementary Data 2.** Temperatures (ºC) by province in China and by country in Europe during 1971-2016. Early [“E”] is March [China] or May [Europe]. Mid [“M”] is April [China] or June [Europe]. Late [“L”] is May [China] or July [Europe]. Winter [“W”] is December to February [China & Europe].

| **year** | **Anhui (E\|M\|L\|W)** | **Gansu (E\|M\|L\|W)** | **Hebei (E\|M\|L\|W)** | **Henan (E\|M\|L\|W)** |
| --- | --- | --- | --- | --- |
| 1973 | 10.2\|17.19\|19.79\|10.65 | 2.85\|10.92\|15.2\|-19.71 | 8.13\|16.23\|20.55\|-5.5 | 9.94\|16.23\|19.84\|7.1 |
| 1974 | 8.89\|17.55\|21.38\|5.75 | 0.31\|11.27\|17.17\|-26.79 | 6.17\|15.13\|21.63\|-5.83 | 7.74\|16.95\|21.48\|2.22 |
| 1975 | 10.14\|15.59\|20.45\|10.2 | 2.98\|9.32\|14.11\|-23 | 8.39\|16.25\|20.88\|-4.42 | 9.9\|15.45\|20.48\|5.28 |
| 1976 | 8.58\|14.27\|21.08\|11.25 | 0.43\|8.79\|15.36\|-21.75 | 5.92\|12.65\|20.88\|-2.06 | 8\|13.59\|21.26\|6.4 |
| 1977 | 10.95\|16.38\|19.02\|8.43 | 2.56\|8.65\|14.74\|-23.33 | 8.29\|15.86\|19.83\|-6.61 | 10.96\|16.81\|19.54\|4.42 |
| 1978 | 8.39\|17.54\|22.51\|11.31 | 1.7\|11.46\|17.23\|-21.14 | 6.15\|15.61\|20.13\|-5.01 | 8.11\|17.3\|21.99\|7.06 |
| 1979 | 9.65\|14.79\|20.04\|13.85 | 0.48\|9.5\|15.23\|-16.03 | 6.43\|11.87\|20.25\|-3.24 | 8.82\|13.5\|19.77\|9.66 |
| 1980 | 6.85\|14.76\|20.96\|9.61 | 1.82\|9.27\|15.76\|-21.66 | 5.25\|12.46\|21.23\|-6.96 | 6.87\|14.37\|21.63\|4.75 |
| 1981 | 11.6\|16.06\|21.98\|8.31 | 4.27\|12.06\|15.8\|-25.07 | 8.57\|16.64\|21.53\|-6.72 | 10.22\|16.3\|22.35\|5.21 |
| 1982 | 8.93\|15.29\|23.1\|9.85 | 3.28\|9.37\|15.11\|-17.88 | 8.05\|16.71\|22.11\|-3.34 | 8.94\|15.85\|22.99\|6.21 |
| 1983 | 8.81\|16.33\|22.44\|10.76 | 1.82\|7.84\|16.21\|-22.97 | 7.44\|15.9\|21.34\|-2.56 | 8.24\|16.3\|21.27\|5.44 |
| 1984 | 9.21\|15.11\|20.31\|3.8 | 1.55\|9.49\|16.6\|-29.47 | 5.15\|14.02\|20.26\|-8.37 | 7.66\|15.2\|20.21\|0.2 |
| 1985 | 6.78\|16.66\|21.03\|6.67 | 0.39\|10.93\|15.02\|-23.18 | 4.6\|15.35\|19.66\|-9.07 | 5.84\|16.19\|19.83\|1.55 |
| 1986 | 9.71\|16.19\|22.23\|10.08 | 1.81\|8.55\|17.63\|-20.14 | 7.52\|15.46\|21.4\|-4.76 | 8.73\|15.95\|21.99\|4.72 |
| 1987 | 7.25\|15.53\|21.32\|13.57 | 2.57\|11.36\|14.89\|-13.21 | 5.74\|14.6\|20.76\|-3 | 6.63\|15.25\|21.12\|9.13 |
| 1988 | 7.14\|16.56\|20.66\|10.72 | 0.41\|10.27\|14.87\|-20.31 | 5.32\|15.84\|20.13\|-2.93 | 6.75\|16.13\|20.6\|5.29 |
| 1989 | 9.62\|15.82\|20.13\|11.04 | 1.88\|10.44\|17.09\|-20.48 | 9.24\|16.11\|21.16\|0.73 | 8.57\|15.88\|20.65\|5.2 |
| 1990 | 10.63\|15.43\|20.8\|8.99 | 4.19\|8.55\|15.77\|-17.03 | 8.07\|14.12\|20.05\|-5.78 | 8.5\|14.91\|20.57\|3.24 |
| 1991 | 7.06\|14.84\|19.37\|11.51 | 3.27\|9.81\|15.56\|-18.79 | 4.45\|14.25\|20.01\|-2.56 | 5.76\|14.34\|19.02\|5.97 |
| 1992 | 7.3\|17.6\|21.95\|13.7 | 2.64\|11.71\|15.13\|-19.12 | 6.95\|16.15\|20.87\|1.41 | 6.59\|16.76\|20.72\|8.03 |
| 1993 | 9.42\|16.17\|19.51\|9.6 | 4.04\|10.39\|14.04\|-18.43 | 8.92\|14.38\|21.18\|-1.07 | 8.88\|15.67\|19.48\|6.78 |
| 1994 | 9.17\|17.29\|23.77\|12.92 | 1.52\|11.45\|16.14\|-17.78 | 6.28\|17.07\|21.81\|-1.83 | 8.17\|16.8\|23.19\|8.49 |
| 1995 | 10.5\|15.2\|21.95\|12.37 | 1.74\|8.47\|16.12\|-22.57 | 8.48\|15.14\|20.18\|2.23 | 9.44\|15.41\|21.91\|9.25 |
| 1996 | 8.32\|15.18\|21.37\|12.21 | 1.96\|9.78\|17.04\|-22.17 | 6.89\|14.72\|21.88\|-0.27 | 7.07\|14.86\|21.28\|7.69 |
| 1997 | 10.7\|16.19\|23.3\|12.7 | 5.96\|11.18\|17.99\|-19.74 | 8.95\|15.36\|21.09\|-2.05 | 10.18\|15.49\|21.66\|7.73 |
| 1998 | 9.3\|18.31\|20.13\|13.12 | 3.38\|13.35\|15.25\|-16.25 | 8.66\|16.16\|20.71\|2.29 | 9.08\|17.22\|19.72\|10.08 |
| 1999 | 8.78\|16.21\|21.2\|15.88 | 2.15\|12.08\|16.68\|-17.18 | 6.2\|16.29\|21.11\|4.29 | 8.5\|16.71\|21.67\|14.25 |
| 2000 | 11.48\|17.07\|22.49\|9.5 | 2.88\|10.47\|17.94\|-20.2 | 9.73\|16.29\|21.74\|-5.35 | 11.88\|17.36\|23.04\|5.31 |
| 2001 | 10.69\|15.07\|22.77\|8.37 | 2.72\|9.98\|16.56\|-20.5 | 9.21\|15.21\|23.79\|-6.39 | 11.75\|15.52\|24.03\|2.32 |
| 2002 | 11.82\|15.57\|19.04\|14.61 | 4.59\|9.84\|15.79\|-16.51 | 10.82\|15.21\|22.48\|4.37 | 12.86\|16.05\|20.25\|13.69 |
| 2003 | 9.14\|14.67\|20.63\|9.3 | 3.21\|9.84\|16.3\|-17.92 | 7.27\|15.95\|21.63\|0.37 | 8.85\|15.77\|21.35\|8.86 |
| 2004 | 10.39\|17.29\|21.22\|14.73 | 3.8\|13.64\|15.93\|-18.13 | 9.1\|16.96\|21\|3.1 | 11.08\|17.98\|22.35\|11.73 |
| 2005 | 9.02\|18.39\|21.23\|4.65 | 3.59\|11.52\|16.77\|-22.84 | 7.47\|17.43\|20.71\|-6.08 | 8.95\|18.66\|21.3\|1.86 |
| 2006 | 11.92\|16.79\|21.48\|9.6 | 2.66\|11.53\|16.1\|-16.45 | 9.62\|14.43\|21.06\|-1.69 | 11.27\|16.58\|21.1\|5.86 |
| 2007 | 10.48\|15.93\|23.04\|14.68 | 2.9\|10.24\|17.81\|-13.48 | 7.39\|15.84\|23.38\|4.19 | 9.68\|16.33\|23.76\|12.89 |
| 2008 | 11.67\|15.58\|21.87\|5.31 | 6.01\|11.94\|18.48\|-27.63 | 10.18\|16.64\|21.46\|-0.28 | 11.66\|16.25\|22.65\|5.12 |
| 2009 | 9.93\|16.34\|20.23\|11.67 | 5.01\|14.53\|16.84\|-14.88 | 8.5\|16.24\|23\|-0.67 | 9.61\|16.25\|20.97\|8.91 |
| 2010 | 8.42\|13.15\|20.78\|13.54 | 1.68\|8.51\|16.26\|-15.42 | 5.32\|12.13\|22.62\|-2.95 | 8.37\|13.24\|21.28\|9.13 |
| 2011 | 9.51\|17.25\|21.22\|6.16 | -0.06\|12.91\|17.26\|-26.13 | 7.77\|14.44\|19.98\|-7.86 | 9.77\|16.41\|20.79\|3.46 |
| 2012 | 8.57\|17.08\|22.18\|6.09 | 3.48\|11.75\|18.24\|-26.23 | 5.8\|16.13\|22.23\|-10.15 | 8.57\|17.5\|22.79\|2.65 |
| 2013 | 11.03\|15.51\|22.16\|9.27 | 7.15\|12.17\|18.28\|-19.82 | 6.99\|12.54\|21.06\|-7.29 | 11.04\|15.73\|22.36\|5.93 |
| 2014 | 11.93\|15.88\|21.81\|11.83 | 4.45\|12.11\|16.65\|-20.92 | 10.22\|16.44\|21.91\|-2.43 | 12.73\|16.56\|23.39\|10.47 |
| 2015 | 10.8\|12.72\|21.61\|14.37 | 3.87\|7.76\|18.09\|-16.12 | 9.22\|12.3\|20.58\|0.04 | 10.89\|12.49\|21.78\|12.79 |
| 2016 | 11.48\|17.51\|20.07\|13.24 | 5.16\|13.08\|16.11\|-19.88 | 9.46\|16.93\|20.46\|-3.07 | 11.86\|18.15\|21.45\|11 |

| **year** | **Hubei (E\|M\|L\|W)** | **Jiangsu (E\|M\|L\|W)** | **Ningxia (E\|M\|L\|W)** | **Shaanxi (E\|M\|L\|W)** |
| --- | --- | --- | --- | --- |
| 1973 | 12.1\|18.18\|20.21\|20.18 | 9.84\|16.78\|19.34\|12.09 | 3.85\|12.46\|16.61\|-16.49 | 4.29\|12.08\|16.33\|-20.67 |
| 1974 | 11.41\|19.41\|22.16\|13.98 | 8.49\|16.41\|20.97\|8.95 | 1.66\|12.29\|18\|-21.91 | 0.85\|11.6\|17.91\|-24.77 |
| 1975 | 11.69\|16.89\|19.99\|19.48 | 9.32\|14.97\|19.66\|11.13 | 3.15\|10.39\|15.65\|-19.44 | 3.28\|9.67\|15.9\|-21.4 |
| 1976 | 9.74\|15.72\|22.13\|19.12 | 8.19\|14.45\|20.6\|12.73 | 2.33\|10.26\|16.27\|-15.82 | 0.77\|9.48\|15.82\|-20.83 |
| 1977 | 13.61\|17.85\|20.16\|16.66 | 9.98\|15.93\|18.6\|8.78 | 3.8\|10.57\|16.4\|-18.55 | 3.16\|10.87\|16.04\|-22.9 |
| 1978 | 11.16\|18.51\|22.32\|19.93 | 8\|16.5\|21.13\|12.04 | 2.32\|12.18\|18.53\|-17.35 | 1.94\|11.33\|17.99\|-22.64 |
| 1979 | 11.24\|16.74\|20.2\|23.44 | 9.35\|14.67\|19.39\|15.42 | 3.03\|11.57\|15.93\|-10.34 | 2.54\|9.9\|15.89\|-14.44 |
| 1980 | 8.5\|16.48\|22.47\|18.9 | 7.17\|14.13\|20.31\|9.18 | 2.71\|10.54\|17.15\|-19.87 | 2.27\|9.29\|16.36\|-24.57 |
| 1981 | 13.29\|17.16\|22.26\|17.56 | 10.6\|15.17\|20.78\|8.8 | 5.63\|11.63\|16.72\|-17.21 | 4.6\|11.26\|16.15\|-21.71 |
| 1982 | 10.27\|16.94\|23.57\|18.75 | 8.8\|14.23\|22.13\|10.73 | 4.39\|11.17\|17.82\|-14.3 | 3.82\|10.8\|17.78\|-18.72 |
| 1983 | 10.97\|17.14\|22.72\|19.35 | 8.35\|15.4\|21.23\|10.56 | 2.73\|10.15\|17.62\|-17.71 | 2.16\|9.99\|17.41\|-21.84 |
| 1984 | 11.23\|17.05\|20.93\|12.29 | 8.72\|14.06\|19.31\|5.36 | 2.93\|11.55\|17.41\|-26.36 | 1.4\|11.24\|16.91\|-29.72 |
| 1985 | 8.77\|18.14\|21.94\|15.94 | 6.71\|15.77\|20.9\|8.62 | 1.27\|12.21\|17.28\|-17.54 | 0.84\|11.45\|17.34\|-21.94 |
| 1986 | 11.82\|16.84\|23.62\|19.68 | 8.97\|14.73\|21.22\|10.5 | 2.16\|10.4\|19.17\|-17.5 | 1.95\|9.07\|18.37\|-21.99 |
| 1987 | 9.76\|17\|21.79\|22.24 | 7.61\|14.65\|20.37\|13.06 | 2.99\|13.16\|17.58\|-10.74 | 2.04\|12.33\|17.41\|-15.04 |
| 1988 | 8.91\|18.49\|22.01\|17.49 | 7.08\|15.8\|20.33\|11.65 | 1.03\|10.93\|17.2\|-16.04 | -0.04\|10.16\|17.01\|-20.72 |
| 1989 | 10.39\|16.69\|21.3\|17.62 | 9.42\|15.04\|19.66\|14.2 | 3.56\|11.18\|17.85\|-15.49 | 1.87\|11.31\|17.68\|-17.58 |
| 1990 | 11.75\|16.43\|21.49\|17.34 | 11.33\|14.74\|20.3\|11.68 | 5.6\|9.61\|17.12\|-12.66 | 4.61\|8.81\|16.62\|-16.38 |
| 1991 | 9.34\|15.68\|20.08\|19.87 | 7.62\|14.2\|19.77\|13.16 | 4.56\|11.02\|16.47\|-12.94 | 4.05\|10.5\|15.79\|-16.98 |
| 1992 | 8.56\|18.93\|22.07\|21.69 | 7.54\|15.91\|21.12\|14.52 | 3.87\|12.48\|17.19\|-14.74 | 3.34\|11.52\|17.19\|-20.11 |
| 1993 | 10.7\|17.62\|20.01\|19.34 | 9.02\|15.41\|19.24\|11.2 | 5.39\|11.05\|16.66\|-19.51 | 4.66\|10.37\|16\|-25.28 |
| 1994 | 10.93\|17.45\|24.05\|19.29 | 9.01\|16.29\|22.74\|14.76 | 2.94\|13.6\|18.23\|-13.47 | 1.96\|12.44\|18.42\|-17.05 |
| 1995 | 12.4\|16.5\|22.61\|20.22 | 10.33\|14.56\|20.6\|12.3 | 4.12\|9.76\|17.41\|-14.65 | 3.06\|9.55\|17.36\|-18.87 |
| 1996 | 9.36\|16.35\|21.46\|20.07 | 7.89\|14.83\|20.35\|11.55 | 2.14\|9.73\|16.91\|-15.44 | 1.54\|8.89\|16.69\|-21.2 |
| 1997 | 12.35\|16.9\|23.32\|20.07 | 10.67\|15.61\|23.07\|13.41 | 6.51\|11.9\|18.58\|-13.24 | 6.32\|11.21\|18.33\|-15.62 |
| 1998 | 10.4\|19.76\|21.67\|21.86 | 9.2\|18.14\|20.49\|15.27 | 4.29\|15.49\|16.37\|-9.96 | 3.92\|15.44\|16.02\|-13.6 |
| 1999 | 10.51\|18.43\|22.16\|26.35 | 9.25\|15.83\|21.53\|17.04 | 4.96\|13.2\|18.59\|-11.18 | 4.59\|12.75\|18.68\|-14.37 |
| 2000 | 13.68\|18.35\|23.99\|18.29 | 10.95\|16.77\|22.33\|12.96 | 4.95\|11.81\|19.76\|-14.88 | 4.72\|10.7\|19.46\|-18.72 |
| 2001 | 13.69\|16.89\|23.21\|18.7 | 10.65\|15.3\|22.35\|14.34 | 4.88\|11.44\|19.03\|-12.49 | 3.94\|11.04\|19\|-15.3 |
| 2002 | 14.38\|16.56\|19.78\|24.37 | 12.12\|16.31\|19.39\|17.46 | 6.35\|11.97\|17.04\|-9.95 | 6.16\|11.19\|17.12\|-10.98 |
| 2003 | 10.86\|16.89\|21.36\|19.47 | 9.54\|15.03\|20.35\|12.9 | 4.72\|12.36\|18.1\|-13.39 | 4.27\|10.87\|17.97\|-16.32 |
| 2004 | 13.38\|20.32\|22.65\|23.35 | 9.93\|16.79\|21.06\|17.81 | 6.12\|15.71\|18.33\|-9.95 | 4.18\|14.94\|17.08\|-12.68 |
| 2005 | 11.56\|20.69\|22.43\|15.22 | 9.18\|18.41\|21.26\|8.6 | 4.49\|14.19\|18.92\|-19.48 | 3.27\|13.08\|17.94\|-24.72 |
| 2006 | 13.62\|19.04\|23.29\|18.55 | 11.37\|16.85\|20.91\|12.92 | 5.42\|14.02\|18.88\|-13.34 | 4.76\|12.65\|17.61\|-15.36 |
| 2007 | 12.98\|17.98\|24.81\|24.37 | 11.42\|16.03\|23.49\|18.4 | 4.82\|12.22\|20.11\|-8.7 | 3.33\|10.94\|18.91\|-9.66 |
| 2008 | 14.5\|17.82\|23.78\|16.19 | 11.32\|15.75\|22.26\|9.2 | 7.6\|13.72\|19.09\|-21.56 | 6.39\|12.38\|17.82\|-23.74 |
| 2009 | 12.42\|17.58\|21.08\|21.37 | 9.78\|16.62\|21.72\|14 | 6.06\|14.77\|18.54\|-10.14 | 4.54\|13.31\|17.46\|-13.67 |
| 2010 | 11.51\|15.92\|21.36\|21.57 | 8.65\|12.66\|21.05\|15.71 | 4.57\|10.06\|17.87\|-11.41 | 2.24\|8.3\|17.1\|-14.15 |
| 2011 | 11.44\|18.67\|22.91\|16.77 | 9.21\|16.75\|21.8\|8.61 | 2.32\|13.97\|17.52\|-16.94 | 1.74\|11.56\|16.6\|-19.93 |
| 2012 | 10.56\|18.4\|22.24\|15.3 | 9.03\|17.95\|21.73\|9.27 | 4.78\|13.71\|19.28\|-17.81 | 3.05\|12.97\|18.3\|-20.85 |
| 2013 | 14.21\|18.13\|22.8\|19.73 | 10.92\|15.91\|21.79\|12.65 | 9.27\|13.33\|19.58\|-9.98 | 7.68\|11.29\|18.82\|-12.27 |
| 2014 | 13.11\|16.74\|20.57\|18.55 | 11.89\|16.27\|22.53\|14.37 | 7.31\|14.14\|18.58\|-11.3 | 6.92\|13.1\|17.55\|-13.13 |
| 2015 | 11.87\|14.39\|21.82\|20.67 | 10.87\|13.27\|21.53\|17.12 | 6.69\|8.93\|18.58\|-7.81 | 5.91\|9.02\|17.92\|-10.26 |
| 2016 | 12.45\|18.19\|19.97\|20.39 | 11.29\|17.53\|20.13\|16.85 | 6.26\|14.43\|17.33\|-12.98 | 5.54\|13.99\|16.57\|-14.71 |

| **year** | **Shandong (E\|M\|L\|W)** | **Shanxi (E\|M\|L\|W)** | **Sichuan (E\|M\|L\|W)** |  |
| --- | --- | --- | --- | --- |
| 1973 | 10.18\|17.24\|20.86\|3.46 | 6.41\|13.47\|18.44\|-9.39 | 13.61\|18.81\|21.03\|23.5 |  |
| 1974 | 7.1\|16.47\|22.53\|0.4 | 2.87\|12.87\|19.31\|-14.19 | 10.51\|18.64\|22.01\|18.13 |  |
| 1975 | 9.94\|16.22\|21.31\|2.78 | 5.11\|11.36\|16.86\|-11.62 | 13.71\|17.08\|19.89\|19.54 |  |
| 1976 | 7.86\|13.71\|21.87\|4.43 | 2.13\|9.81\|17.68\|-9.97 | 9.87\|15.69\|20.33\|20.43 |  |
| 1977 | 11.14\|17.19\|20.2\|3.17 | 5.91\|12.88\|17.42\|-12.63 | 12.41\|17.14\|20.16\|18.06 |  |
| 1978 | 8.34\|17.82\|23.03\|4.36 | 4\|13.34\|18.61\|-11.5 | 11.84\|17.56\|21.82\|20.63 |  |
| 1979 | 8.9\|13.22\|21.67\|7.52 | 4.05\|10.28\|17.21\|-6.97 | 11.58\|16.9\|19.15\|24.82 |  |
| 1980 | 6.73\|14.06\|21.76\|0.69 | 3.72\|11.24\|18.74\|-12.23 | 11.35\|16.89\|20.9\|19.68 |  |
| 1981 | 10.85\|17.77\|22.5\|1.75 | 6.99\|13.6\|17.75\|-10.91 | 12.98\|17.33\|20.28\|19.78 |  |
| 1982 | 9.97\|16.84\|24.25\|4.43 | 5.89\|12.87\|19.34\|-8.08 | 11.98\|14.82\|22.16\|19.45 |  |
| 1983 | 8.26\|17.11\|22.75\|4.66 | 4.35\|12.39\|18.57\|-10.88 | 10\|16.12\|21.15\|19.65 |  |
| 1984 | 6.7\|15.89\|21.3\|-2.42 | 3.16\|12.26\|17.2\|-16.61 | 12\|17.06\|20.3\|14.15 |  |
| 1985 | 6.12\|16.48\|19.96\|-1.02 | 2.7\|13.94\|17.49\|-12.09 | 8.75\|16.63\|22.01\|19.81 |  |
| 1986 | 9.42\|16.6\|22.98\|2.41 | 5.01\|12.1\|19.22\|-11.24 | 11.07\|15.82\|21.73\|20.64 |  |
| 1987 | 7.06\|16.11\|21.95\|6.69 | 3.72\|13.25\|18.89\|-7.12 | 12.65\|16.81\|21.74\|24.28 |  |
| 1988 | 6.89\|16.78\|21.32\|4.33 | 2.01\|11.44\|17.88\|-10.47 | 8.52\|16.3\|21.53\|20.22 |  |
| 1989 | 9.12\|17.23\|22.65\|6.69 | 4.38\|13.31\|18.44\|-8.13 | 11.33\|15.84\|20.41\|18.86 |  |
| 1990 | 9.18\|15.36\|20.79\|3.19 | 5.26\|10.63\|17.93\|-10.47 | 13.01\|15.35\|20.73\|21.21 |  |
| 1991 | 5.95\|15.78\|20.45\|5.42 | 3.2\|11.9\|16.74\|-8.99 | 12.7\|16.17\|20.34\|21.3 |  |
| 1992 | 7.49\|17.03\|21.86\|7.87 | 4.3\|13.59\|18.07\|-9.84 | 9.97\|17.8\|20.64\|20.68 |  |
| 1993 | 9.45\|15.84\|21.18\|5.86 | 6.18\|12.41\|16.69\|-12.05 | 11.98\|17.62\|20.35\|20.5 |  |
| 1994 | 7.81\|18.63\|23.12\|6.89 | 3.85\|13.73\|19.52\|-7.27 | 10.37\|18.09\|23.25\|22.39 |  |
| 1995 | 9.5\|16.3\|21.9\|7.73 | 4.88\|12.05\|18.39\|-8.07 | 11.23\|16.35\|22.37\|20.64 |  |
| 1996 | 7.6\|15.26\|22.69\|7.06 | 3.66\|10.97\|18.09\|-10.18 | 10.58\|15.04\|19.93\|18.6 |  |
| 1997 | 10.52\|16.05\|21.58\|6.55 | 7.38\|12.7\|19.34\|-7.27 | 13.64\|17.13\|22.71\|21.65 |  |
| 1998 | 9.63\|18.17\|20.8\|8.13 | 5.7\|15.79\|17.83\|-5.85 | 11.71\|22.21\|21.94\|24.2 |  |
| 1999 | 7.97\|16.94\|22.06\|10.07 | 6.07\|14.47\|19.62\|-5.26 | 12.96\|19.19\|21.11\|25.47 |  |
| 2000 | 10.89\|16.75\|21.55\|1.55 | 6.98\|12.69\|20.58\|-9.67 | 13.05\|17.39\|24.32\|20.8 |  |
| 2001 | 9.8\|15.85\|24.8\|0.05 | 6.64\|13.04\|20.56\|-8.45 | 15.2\|17.32\|23.26\|23.54 |  |
| 2002 | 12.1\|15.74\|20.77\|9.32 | 8.44\|13.21\|18.58\|-2.88 | 14.15\|17.4\|21.08\|23.45 |  |
| 2003 | 7.96\|15.49\|21.47\|3.78 | 5.28\|11.42\|18.81\|-7.7 | 12.94\|17.67\|21.56\|25.78 |  |
| 2004 | 10.46\|17.72\|20.93\|7.62 | 5.67\|14.46\|17.82\|-7.49 | 13.19\|19.47\|21.41\|24.93 |  |
| 2005 | 7.35\|17.97\|21.61\|-3.18 | 4.78\|14.03\|19.68\|-13.96 | 12.51\|19.52\|22.22\|19.61 |  |
| 2006 | 10.51\|16.48\|21.61\|3.97 | 6.89\|14.29\|18.63\|-6.33 | 13.51\|19.21\|22.71\|23.02 |  |
| 2007 | 9.09\|16.06\|23.36\|9.69 | 6.69\|13.68\|20.99\|-0.69 | 14.11\|17.99\|24.35\|28.07 |  |
| 2008 | 10.42\|16.5\|22.31\|1.88 | 7.39\|13.33\|18.9\|-10.87 | 14.39\|18.31\|23.23\|19.13 |  |
| 2009 | 9.13\|17\|21.91\|6.21 | 6.15\|14.49\|18.44\|-8.68 | 13.84\|18.76\|22.27\|27.47 |  |
| 2010 | 6.36\|12.56\|22.39\|4.75 | 3.89\|9.47\|18.94\|-7 | 13.42\|15.55\|21.04\|26.79 |  |
| 2011 | 8.94\|15.53\|20.96\|-0.12 | 3.99\|13.18\|17.92\|-13.37 | 10.9\|19.28\|21.99\|20.45 |  |
| 2012 | 7.33\|17.65\|23.84\|-1.09 | 4.41\|14.37\|20.55\|-14.24 | 12.75\|19.52\|22.44\|20.64 |  |
| 2013 | 9.6\|14.16\|22.18\|2.6 | 8.44\|12.08\|20.39\|-7.76 | 18.06\|19.67\|22.52\|25.59 |  |
| 2014 | 12.65\|17.16\|24.11\|6.69 | 8.36\|14.1\|19.43\|-7.97 | 13.69\|19.65\|22.34\|24.19 |  |
| 2015 | 10.92\|10.77\|21.72\|8.95 | 7.44\|10.57\|19.1\|-4.04 | 15.46\|20.94\|24\|28.03 |  |
| 2016 | 11.46\|18.77\|21.21\|6.17 | 6.58\|14.61\|18.45\|-7.31 | 14.87\|19.24\|22.34\|27.04 |  |

| **Year** | **Belgium (E\|M\|L\|W)** | **Czech (E\|M\|L\|W)** | **Denmark (E\|M\|L\|W)** | **France (E\|M\|L\|W)** |
| --- | --- | --- | --- | --- |
| 1971 | 14.3\|14.21\|18.52\|10.35 | 14.08\|14.24\|18.15\|-1.28 | 11.75\|13.5\|16.55\|6.82 | 13.24\|15\|19.35\|10.62 |
| 1972 | 11.73\|13.89\|17.52\|9.28 | 12.08\|15.75\|18.28\|-3.92 | 10.91\|13.9\|17.47\|0.92 | 11.92\|14.76\|17.91\|11.27 |
| 1973 | 12.63\|16.7\|17.38\|7.69 | 12.84\|15.66\|17.31\|-2.75 | 10.9\|15.18\|17.71\|4.75 | 13.39\|17.02\|18.24\|9.14 |
| 1974 | 11.75\|15.08\|15.86\|16.67 | 11.41\|13.79\|15.8\|6.4 | 10.54\|14.31\|14.61\|10.2 | 12.32\|15.83\|18.14\|16.84 |
| 1975 | 11.41\|15.16\|18.3\|12.07 | 12.93\|15.04\|18.13\|1.22 | 10.87\|14.32\|17.23\|9.44 | 11.96\|15.72\|18.96\|13.47 |
| 1976 | 13.78\|18.84\|20.17\|7.77 | 12.77\|16.64\|19.02\|-3.82 | 11.17\|15.02\|18.17\|-0.31 | 13.57\|18.86\|19.97\|12.46 |
| 1977 | 11.86\|14.48\|16.97\|12.6 | 12.19\|16.24\|16.64\|-1.28 | 11.03\|14.92\|15.61\|1.77 | 12.53\|15.28\|17.84\|14.87 |
| 1978 | 12.12\|14.84\|15.72\|6.69 | 11.31\|14.61\|15.53\|-4.5 | 11.57\|14.96\|14.91\|-1.86 | 12.23\|15.18\|17.43\|10.97 |
| 1979 | 11.88\|15.48\|16.59\|3.3 | 13.34\|17.82\|15.04\|-5.05 | 10.59\|14.45\|14.24\|-6.09 | 12.32\|16.37\|18.6\|9.71 |
| 1980 | 11.76\|14.69\|15.53\|8.38 | 9.98\|14.83\|15.41\|-5.59 | 10.65\|14.98\|16.21\|-1.57 | 11.86\|15.15\|17.05\|10.76 |
| 1981 | 12.99\|14.73\|16.63\|4.54 | 13.22\|16.34\|16.66\|-8 | 12.62\|13.67\|15.73\|-4.4 | 12.34\|16.19\|17.6\|10.63 |
| 1982 | 12.93\|16.73\|18.87\|7.93 | 13.01\|16.36\|18.61\|-6.14 | 10.72\|14.53\|17.54\|-2.44 | 13.18\|17.25\|19.9\|13.52 |
| 1983 | 10.7\|16.44\|20.95\|9.55 | 13.1\|16.09\|20.57\|-1.87 | 10.53\|14.52\|17.69\|4.7 | 11.45\|17.01\|21.91\|10.58 |
| 1984 | 10.16\|14.26\|16.79\|9.25 | 11.67\|14.01\|15.65\|-3.31 | 11.32\|13.41\|15.41\|2.5 | 10.37\|15.92\|18.97\|11.82 |
| 1985 | 13.02\|13.99\|17.9\|1.85 | 13.82\|13.39\|17.57\|-11.16 | 11.49\|13.29\|16.15\|-7.69 | 12.55\|15.13\|19.19\|6.64 |
| 1986 | 13.36\|16.81\|17.53\|3.07 | 14.73\|15.57\|17\|-9.58 | 11.46\|14.79\|15.89\|-4.78 | 13.59\|17.01\|18.98\|9.48 |
| 1987 | 10.23\|14.12\|17.21\|2.38 | 10.51\|14.82\|17.76\|-8.89 | 9.22\|11.89\|15.22\|-3.59 | 11.65\|15.45\|18.6\|9.06 |
| 1988 | 14.1\|15.03\|16.25\|16.01 | 14.2\|15.27\|18.07\|3.83 | 12.36\|15.79\|16.57\|8.74 | 13.83\|16.42\|17.9\|15.73 |
| 1989 | 15.01\|15.81\|18.69\|13.03 | 13.24\|14.74\|17.9\|3.11 | 11.88\|15.11\|17.09\|10.85 | 15.17\|16.62\|20\|13.62 |
| 1990 | 14.64\|15.11\|17.65\|14.92 | 13.75\|15.7\|16.98\|2.9 | 12.67\|14.91\|16.1\|11.38 | 14.95\|16.41\|19.41\|15.57 |
| 1991 | 10.31\|13.29\|18.99\|5.57 | 9.34\|14.56\|19.14\|-6.51 | 9.49\|12.02\|17.93\|3.08 | 11.75\|15.66\|19.59\|10.89 |
| 1992 | 15.16\|16.66\|18.38\|10.27 | 13.69\|17.44\|19.27\|-0.45 | 13.32\|17.59\|17.56\|8.18 | 14.21\|15.29\|18.62\|12.24 |
| 1993 | 14.42\|16.57\|16.87\|11.49 | 15.44\|15.78\|16.54\|-0.84 | 13.03\|13.91\|14.64\|4.51 | 13.18\|17.05\|17.63\|17.86 |
| 1994 | 12.64\|16.14\|21.47\|12.35 | 12.72\|16.34\|21.52\|1.93 | 11.2\|13.27\|19.85\|4.06 | 13.12\|16.01\|20.23\|16.95 |
| 1995 | 13.24\|14.9\|20.77\|10.66 | 12.36\|14.62\|20.34\|-1.14 | 10.16\|13.99\|17.71\|2.95 | 13.01\|16\|20.81\|18.8 |
| 1996 | 10.82\|16.21\|17.02\|2.45 | 12.57\|16.14\|15.9\|-14.13 | 8.72\|13.53\|14.95\|-7.19 | 12.74\|16.83\|18.61\|14.59 |
| 1997 | 13.02\|15.95\|17.55\|9.58 | 13.25\|16.08\|16.86\|-1.96 | 9.86\|15.05\|17.78\|2.6 | 13.82\|16.69\|18.36\|15.12 |
| 1998 | 15.05\|16.23\|16.62\|13.72 | 13.7\|17.07\|17.43\|1.72 | 12.06\|14.15\|14.71\|7.04 | 15.21\|17.25\|18.9\|18.99 |
| 1999 | 14.65\|15.55\|19.59\|11.93 | 13.59\|15.49\|18.93\|-1.49 | 10.8\|13.87\|17.63\|4.17 | 14.67\|16.57\|19.87\|14.76 |
| 2000 | 14.75\|16.77\|15.52\|14.51 | 15.01\|17.5\|15.92\|1.56 | 12.69\|14.04\|15.03\|8.86 | 14.21\|17.51\|17.98\|18.05 |
| 2001 | 14.67\|15.43\|18.82\|9.83 | 14.53\|14.39\|18.52\|-3.92 | 11.54\|13.01\|17.88\|1.88 | 14.84\|16.14\|19.14\|15.79 |
| 2002 | 13.24\|17.13\|17.75\|14.57 | 15.35\|17.59\|18.95\|0.17 | 13.05\|15.92\|17.45\|6.07 | 13.62\|18.38\|18.57\|21.62 |
| 2003 | 13.75\|19.86\|19.51\|8.21 | 15.32\|19.61\|18.98\|-6.17 | 11.64\|16.12\|18.55\|1.71 | 14.2\|20.06\|20.71\|13.71 |
| 2004 | 12.49\|16.16\|17.39\|10.24 | 11.69\|15.51\|17.57\|-2.82 | 11.63\|13.94\|15.77\|4.53 | 13.33\|17.31\|18.82\|15.93 |
| 2005 | 13.05\|17.87\|18.39\|9.29 | 13.46\|16.24\|18.32\|-4.61 | 11.12\|14.26\|17.76\|5.11 | 14.87\|18.04\|19.16\|17.11 |
| 2006 | 13.84\|17.22\|21.2\|8.77 | 12.99\|17.18\|21.39\|-5.75 | 11.78\|15.52\|20.57\|5.23 | 14.46\|18.34\|21.16\|18.22 |
| 2007 | 14.4\|17.34\|17.18\|16.45 | 14.57\|18.37\|18.9\|5.68 | 11.94\|16.51\|16.2\|10.2 | 14.61\|17.36\|18.24\|24.95 |
| 2008 | 15.96\|16.37\|18.09\|13.67 | 14.09\|17.85\|18.45\|4.88 | 12.98\|15.49\|18.03\|10.46 | 15.49\|16.47\|19.09\|24.46 |
| 2009 | 14.25\|16.18\|18.55\|5.83 | 13.98\|15.19\|18.79\|-5.04 | 11.98\|14.28\|17.83\|1.37 | 15.14\|17.14\|19.59\|18.96 |
| 2010 | 10.99\|17.15\|19.28\|0.71 | 11.81\|16.87\|20.58\|-11.26 | 9.7\|14.39\|19.37\|-10.73 | 12.48\|16.87\|19.72\|14.53 |
| 2011 | 14.67\|16.73\|16.38\|13.8 | 13.92\|17.38\|16.97\|-0.34 | 11.92\|15.64\|16.97\|4.02 | 15.39\|17.1\|17.79\|24.64 |
| 2012 | 14.15\|15.39\|17.32\|9.6 | 14.98\|17.23\|18.9\|-5.78 | 12.64\|13.38\|16.68\|1.47 | 14.3\|17.21\|18.45\|21.57 |
| 2013 | 10.99\|15.57\|20.08\|8.33 | 12.54\|16.27\|20.24\|-1.22 | 12.48\|14.73\|18.25\|4.58 | 12.22\|16.62\|21.6\|19.38 |
| 2014 | 13.29\|16.7\|19.31\|16.22 | 12.57\|16.74\|19.92\|4.94 | 12.31\|15.62\|20\|9.22 | 13.64\|17.73\|18.42\|25.62 |
| 2015 | 12.82\|16.51\|19.19\|14.84 | 12.92\|16.5\|20.77\|5.28 | 9.99\|13.15\|16.05\|10.58 | 14.12\|18.35\|21.11\|15.7 |

| **Year** | **Germany (E\|M\|L\|W)** | **Netherlands (E\|M\|L\|W)** | **Norway (E\|M\|L\|W)** | **Serbia (E\|M\|L\|W)** |
| --- | --- | --- | --- | --- |
| 1971 | 13.91\|13.99\|18.2\|3.83 | 13.54\|13.82\|17.45\|11.73 | 4.93\|9.01\|11.19\|-18.62 | 18.83\|19.63\|20.9\|2.62 |
| 1972 | 11.47\|14.59\|17.83\|0.25 | 11.69\|13.66\|17.32\|7.78 | 5.32\|10.95\|13.78\|-18.94 | 17.37\|21.67\|21.8\|2.8 |
| 1973 | 12.6\|16.2\|17.45\|1.29 | 11.99\|15.76\|17\|9.33 | 4.44\|9.88\|13.81\|-20.14 | 17.47\|19.8\|21.67\|2.05 |
| 1974 | 11.07\|14.24\|15.56\|10.93 | 11.61\|14.52\|15.19\|16.14 | 5.37\|10.31\|11.4\|-14.07 | 15\|18.53\|20.5\|4.47 |
| 1975 | 11.8\|14.7\|18.35\|7.1 | 10.87\|14.58\|17.65\|13.24 | 4.67\|7.77\|11.3\|-17.15 | 17.93\|19.53\|21.23\|2.25 |
| 1976 | 12.9\|17.39\|19.33\|0.6 | 13.11\|17.49\|19.19\|8.06 | 6.1\|9.11\|12.02\|-26.7 | 15.97\|18.03\|21.2\|0.58 |
| 1977 | 11.7\|15.45\|16.55\|5.43 | 11.65\|14.15\|16.58\|12.36 | 3.94\|8.35\|11.36\|-26.4 | 17.5\|19.87\|21.3\|5.2 |
| 1978 | 11.54\|14.86\|15.66\|-0.21 | 11.94\|14.61\|15.26\|5.9 | 5.1\|10.26\|11.79\|-32.44 | 14.97\|18.83\|20.3\|1.83 |
| 1979 | 12.55\|16.51\|15.49\|-2.64 | 11.54\|14.86\|15.74\|1.74 | 3.6\|9.99\|11.5\|-30.49 | 16.93\|21.57\|19.77\|1.67 |
| 1980 | 10.64\|14.72\|15.27\|0.18 | 11.76\|14.52\|15.52\|8.35 | 5.04\|11.47\|13.01\|-30.33 | 14.3\|19.33\|20.5\|0.25 |
| 1981 | 13.26\|15.37\|16.51\|-3.71 | 13.27\|14.34\|16.34\|4.25 | 6.09\|7.33\|11.12\|-31.69 | 16.3\|20.53\|20.8\|-0.05 |
| 1982 | 12.57\|16.4\|19.04\|-0.17 | 12.52\|16.08\|18.53\|7.48 | 4.22\|7.72\|12.37\|-23.77 | 17.87\|20.87\|21.27\|-0.1 |
| 1983 | 11.49\|16.3\|20.47\|3.15 | 10.9\|15.89\|19.71\|9.87 | 5.53\|8.74\|12.03\|-21.46 | 18.43\|18.87\|22.6\|2.98 |
| 1984 | 10.99\|13.87\|16.06\|1.97 | 10.64\|13.53\|16.02\|9.65 | 6.99\|9.49\|11.02\|-19.56 | 16.7\|18.4\|19.57\|1.72 |
| 1985 | 13.27\|13.73\|17.68\|-4.53 | 13.07\|13.49\|17.11\|2.96 | 4.56\|9.24\|12.35\|-36.76 | 18.6\|17.9\|21.57\|-3.95 |
| 1986 | 14.24\|15.96\|17.31\|-4.13 | 12.92\|15.77\|16.99\|3.72 | 5.7\|11.01\|11.47\|-32.56 | 18.67\|19.27\|19.73\|0.55 |
| 1987 | 9.84\|13.98\|17.1\|-3.98 | 10.02\|13.54\|16.88\|3.95 | 3.72\|8.11\|11.19\|-30.46 | 15.23\|20.47\|24.2\|-0.22 |
| 1988 | 14.3\|15.22\|17.34\|8.98 | 14.18\|14.63\|16.02\|16.21 | 5.63\|11.01\|13.15\|-20.84 | 17.07\|19.7\|23.77\|4.7 |
| 1989 | 14.02\|15.57\|18\|8.5 | 14.19\|15.45\|17.86\|13.42 | 4.99\|10.1\|11.28\|-14.47 | 15.77\|18.13\|21.8\|2.88 |
| 1990 | 14.14\|15.39\|17.03\|9.4 | 13.72\|14.74\|16.8\|15.99 | 5\|10.42\|12.03\|-11.61 | 17.17\|19.97\|21.77\|3.8 |
| 1991 | 10.07\|13.92\|19.64\|1.42 | 9.87\|12.49\|18.87\|6.55 | 3.79\|8.35\|12.8\|-19.13 | 13.27\|20.37\|22.07\|0.37 |
| 1992 | 15\|17.42\|19.12\|5.39 | 15.22\|16.88\|18.2\|11.63 | 6.42\|11.68\|10.93\|-12.27 | 16.87\|20.3\|22.07\|2.35 |
| 1993 | 14.77\|16.12\|16.53\|5.52 | 14.15\|15.56\|16.04\|11.92 | 5.71\|7.47\|11.54\|-18.62 | 19\|20.8\|22.07\|-0.25 |
| 1994 | 12.71\|16.3\|21.74\|7.22 | 12.25\|14.96\|20.79\|11.97 | 4.19\|7.93\|13.47\|-24.45 | 17.7\|20.3\|23.83\|3.72 |
| 1995 | 12.46\|14.74\|20.53\|4.33 | 12.72\|14.29\|19.95\|9.37 | 3.89\|9.53\|11.13\|-21.58 | 16.23\|20.43\|24.03\|4 |
| 1996 | 11.17\|15.7\|16.31\|-5.98 | 10.48\|15.25\|16.23\|0.24 | 2.76\|8.74\|10.76\|-26.39 | 18.8\|21.17\|21.43\|-0.35 |
| 1997 | 12.75\|16.18\|17.58\|3.99 | 12.5\|15.58\|17.51\|9.79 | 3.66\|10.47\|13.95\|-19.06 | 17.9\|21.13\|21.1\|2.42 |
| 1998 | 14.47\|16.6\|16.64\|9.35 | 14.53\|15.59\|16.11\|15.28 | 5.17\|8.76\|12.03\|-19.16 | 16.4\|22.1\|23.03\|5.2 |
| 1999 | 13.83\|15.66\|19.44\|5.89 | 13.95\|14.91\|19.1\|12.82 | 3.91\|10.17\|11.99\|-24.7 | 17.27\|20.63\|21.9\|1.9 |
| 2000 | 14.82\|17.28\|15.66\|8.7 | 14.46\|15.84\|15.31\|15.28 | 6.04\|8.37\|11.7\|-17.09 | 19.03\|21.7\|22.87\|1.57 |
| 2001 | 14.7\|14.38\|18.96\|3.31 | 13.82\|14.65\|18.47\|10.13 | 4.6\|9.46\|12.13\|-23.49 | 18.27\|18.77\|23.03\|4.62 |
| 2002 | 14.16\|17.66\|17.89\|7.47 | 13.31\|16.46\|17.53\|14.05 | 7.2\|11.95\|13.4\|-22.33 | 19.33\|21.93\|24.1\|4.35 |
| 2003 | 14.54\|21.16\|19.27\|0.62 | 13.25\|17.92\|18.94\|8.42 | 5.24\|10.2\|14.89\|-22.19 | 20.83\|24.17\|23.1\|-1.3 |
| 2004 | 11.7\|15.34\|16.91\|4.31 | 12.19\|15.29\|16.69\|11.3 | 5.66\|8.88\|12.65\|-20.21 | 15.67\|20.23\|22.47\|1.4 |
| 2005 | 13.29\|16.75\|18.5\|2.34 | 12.47\|16.35\|17.71\|11.26 | 3.97\|9.49\|13.64\|-17.93 | 17.2\|19.57\|22.3\|-0.4 |
| 2006 | 13.26\|17.28\|22.44\|2.26 | 13.96\|16.32\|21.78\|10.54 | 6.12\|10.08\|13.49\|-15.46 | 17.07\|20.07\|23.8\|0.5 |
| 2007 | 14.66\|17.82\|17.75\|10.5 | 13.83\|17.04\|16.93\|16.88 | 4.84\|10.98\|12.8\|-20.53 | 19.1\|22.97\|24.9\|7.02 |
| 2008 | 14.78\|17.44\|18.54\|9.46 | 15.33\|16.23\|18.07\|14.18 | 5.38\|9.94\|13.19\|-13.92 | 18.57\|22.43\|22.83\|4.15 |
| 2009 | 14.26\|15.06\|18.63\|0.48 | 13.6\|15.3\|18.05\|6.48 | 6.37\|9.58\|12.67\|-23.61 | 18.97\|20.53\|23.17\|1.43 |
| 2010 | 10.64\|16.61\|20.79\|-7.17 | 10.32\|16.07\|19.74\|-0.58 | 5.03\|8.66\|12.93\|-33.96 | 17.7\|21\|23.7\|1.95 |
| 2011 | 14.24\|17.09\|16.66\|6.69 | 14\|15.96\|15.89\|13.75 | 5.82\|10.81\|12.92\|-21.17 | 17.07\|21.37\|23.27\|0.93 |
| 2012 | 14.45\|16\|17.72\|1.72 | 14.04\|14.64\|17.14\|9.96 | 4.94\|8.76\|11.56\|-24.29 | 17.37\|23.47\|26.07\|-1.12 |
| 2013 | 12.29\|15.94\|20.06\|3.94 | 11.34\|14.89\|19.11\|9.5 | 7.69\|10.84\|12.69\|-18.92 | 18.63\|20.7\|23.03\|3.97 |
| 2014 | 12.91\|16.47\|19.81\|9.63 | 13.14\|15.94\|19.67\|16.59 | 5.35\|9.86\|15.31\|-16.67 | 16.67\|20.7\|22.83\|6.22 |
| 2015 | 12.66\|16.08\|19.76\|9.68 | 12.15\|15.25\|18.35\|16.76 | 4.44\|8.14\|11.14\|-15.22 | 18.47\|20.87\|25.43\|3.53 |

| **Year** | **Sweden (E\|M\|L\|W)** | **UK (E\|M\|L\|W)** |  |  |
| --- | --- | --- | --- | --- |
| 1971 | 6.43\|11.01\|13.31\|-19.31 | 10.27\|11.27\|15.12\|14.2 |  |  |
| 1972 | 5.81\|12.64\|15.81\|-21.28 | 9.52\|10.73\|14.1\|11.82 |  |  |
| 1973 | 6.2\|12.5\|16.27\|-20.83 | 9.89\|13.41\|14.31\|11.48 |  |  |
| 1974 | 6.6\|12.09\|12.76\|-15.23 | 9.9\|12.37\|13.56\|16.39 |  |  |
| 1975 | 6.87\|10.2\|13.7\|-15.36 | 8.79\|13.16\|15.56\|14.05 |  |  |
| 1976 | 7.65\|11.15\|13.54\|-28.66 | 10.38\|14.88\|16.49\|10.02 |  |  |
| 1977 | 5.44\|10.66\|12.33\|-27.55 | 9.35\|11.39\|14.64\|11 |  |  |
| 1978 | 6.36\|11.96\|13.13\|-35.83 | 10.5\|12.42\|13.34\|7.37 |  |  |
| 1979 | 6.12\|12.68\|13.01\|-33.94 | 8.43\|12.77\|14.39\|5.11 |  |  |
| 1980 | 6.19\|13.44\|14.67\|-34.16 | 10.09\|12.63\|13.37\|11.12 |  |  |
| 1981 | 7.88\|9.68\|13.29\|-32.64 | 10.32\|12.12\|14.18\|6.5 |  |  |
| 1982 | 5.86\|9.3\|14.24\|-26.2 | 10.1\|13.68\|15.25\|9.47 |  |  |
| 1983 | 7.34\|10.85\|14.12\|-21.79 | 8.98\|12.72\|17.12\|11.56 |  |  |
| 1984 | 8.91\|11.34\|12.91\|-21.87 | 9.07\|13.23\|15.35\|9.82 |  |  |
| 1985 | 5.54\|11.21\|13.58\|-45.45 | 9.68\|11.47\|14.56\|7.52 |  |  |
| 1986 | 7.61\|13.4\|13.33\|-35.33 | 9.89\|13.29\|14.27\|6.23 |  |  |
| 1987 | 5.09\|9.71\|12.62\|-34.51 | 9.15\|11.39\|14.56\|8.26 |  |  |
| 1988 | 7.48\|13.3\|15.24\|-21.88 | 10.59\|13.45\|13.65\|14.5 |  |  |
| 1989 | 7.44\|12.11\|13.58\|-14.27 | 11.42\|13.06\|16.37\|14.14 |  |  |
| 1990 | 7.49\|12.03\|13.41\|-12.56 | 11.38\|12.53\|15.2\|14.71 |  |  |
| 1991 | 5.09\|9.4\|14.71\|-20.33 | 10.1\|11.1\|15.95\|8.46 |  |  |
| 1992 | 8.62\|13.73\|12.84\|-11.58 | 11.87\|14.35\|14.71\|11.09 |  |  |
| 1993 | 8.05\|9.23\|13.03\|-19.28 | 10.13\|13.26\|13.79\|13.49 |  |  |
| 1994 | 5.65\|10\|16.12\|-27.67 | 9.44\|12.96\|15.95\|12.1 |  |  |
| 1995 | 5.1\|11.69\|12.89\|-22.8 | 10.35\|13.14\|16.6\|11.13 |  |  |
| 1996 | 4.49\|10.8\|12.51\|-28.64 | 8.24\|13.07\|14.84\|9.35 |  |  |
| 1997 | 5.08\|12.69\|15.97\|-18.93 | 10.29\|12.77\|15.34\|13.03 |  |  |
| 1998 | 6.34\|10.35\|13.3\|-19.41 | 11.49\|12.68\|14.16\|16.02 |  |  |
| 1999 | 5.48\|12.4\|14.17\|-27.19 | 11.45\|12.54\|15.83\|12.27 |  |  |
| 2000 | 8.04\|10.55\|13.22\|-16.65 | 10.89\|12.94\|14.15\|14.02 |  |  |
| 2001 | 6.4\|11.58\|14.29\|-25.26 | 11.31\|12.58\|15.18\|9.7 |  |  |
| 2002 | 8.91\|14.02\|15.33\|-23.71 | 10.84\|13.27\|14.34\|15.62 |  |  |
| 2003 | 7.03\|11.93\|16.76\|-24.84 | 10.78\|14.43\|16.18\|11.79 |  |  |
| 2004 | 7.27\|10.7\|13.66\|-22.11 | 11.04\|14.04\|14.49\|13.81 |  |  |
| 2005 | 5.84\|11.38\|15.54\|-19.96 | 10.02\|14.05\|15.24\|13.45 |  |  |
| 2006 | 7.41\|12.49\|15.62\|-17.92 | 10.63\|14.4\|17.39\|13.22 |  |  |
| 2007 | 6.83\|12.85\|14.02\|-21.54 | 10.7\|13.75\|14.39\|15.59 |  |  |
| 2008 | 7.12\|11.97\|14.78\|-14.01 | 12.23\|13.06\|15.38\|13.55 |  |  |
| 2009 | 8.17\|11\|14.22\|-26.63 | 10.99\|13.85\|15.41\|9.43 |  |  |
| 2010 | 6.84\|10.77\|15.51\|-39.53 | 9.83\|14.17\|15.62\|2.99 |  |  |
| 2011 | 7.33\|13.57\|15.47\|-24.48 | 11.17\|12.84\|14.14\|13.34 |  |  |
| 2012 | 6.72\|10.2\|13.53\|-29.85 | 10.46\|12.35\|14.06\|12.92 |  |  |
| 2013 | 9.75\|12.98\|14.09\|-20.66 | 9.84\|13.29\|16.62\|12.17 |  |  |
| 2014 | 6.82\|11.29\|17.29\|-18.58 | 11.22\|14.32\|16.44\|14.47 |  |  |
| 2015 | 5.86\|9.85\|12.74\|-15.84 | 9.72\|12.8\|14.5\|14.38 |  |  |
