# Supplementary material for: Multidecadal, continent-level analysis indicates agricultural practices impact wheat aphid loads more than climate change
Source: Commun Biol. 2022 Jul 28;5:761. doi: 10.1038/s42003-022-03731-z (PMC9334390; doi:10.1038/s42003-022-03731-z)
Supplement: Supplementary file 6 — Supplementary Data 3 [file 42003_2022_3731_MOESM6_ESM.docx]

**Supplementary Data 3.** Data details for land-use intensity.

Crop categories for European countries in FAOSTAT:

almonds with shell, anise, badian, fennel, coriander, apples, apricots, artichokes, asparagus, avocados, barley, beans (dry), beans (green), berries nes, blueberries, broad beans, horse beans, buckwheat, cabbages and other brassicas, canary seed, carrots and turnips, cauliflowers and broccoli, cereals, cherries, cherries (sour), chestnut, chick peas, chicory roots, chillies and peppers (dry), chillies and peppers (green), coconuts, cow peas (dry), cranberries, cucumbers and gherkins, currants, eggplants (aubergines), figs, flax fibre and tow, fruit (citrus), fruit (fresh), fruit (stone), fruit - tropical (fresh), garlic, gooseberries, grain (mixed), grapefruit (inc. pomelos), grapes, hazelnuts (with shell), hemp tow waste, hempseed, hops, kiwi fruit, leeks & other alliaceous vegetables, lemons and limes, lentils, lettuce and chicory, linseed, lupins, maize, maize (green), melons - other (inc.cantaloupes), millet, mushrooms and truffles, mustard seed, nuts, oats, oilseeds, olives, onions (dry), onions & shallots (green), oranges, peaches and nectarines, pears, peas (dry), peas (green), plums and sloes, poppy seed, potatoes, pulses, pumpkins, squash, gourds, quinces, rapeseed, raspberries, rice (paddy), roots and tubers, rye, sorghum, soybeans, spinach, strawberries, string beans, sugar beet, sunflower seed, tangerines, mandarins, clementines, satsumas, tobacco (unmanufactured), tomatoes, triticale, vanilla, vegetables (fresh), vegetables (leguminous), vetches, walnuts (with shell), watermelons, wheat, yams

Crop categories for Chinese provinces in the China Rural Statistical Yearbooks: corn, cotton, peanut, rapeseed, rice, soybeans, total grain, tubers, wheat along with total sown area.
