# Supplementary material for: Multidecadal, continent-level analysis indicates agricultural practices impact wheat aphid loads more than climate change
Source: Commun Biol. 2022 Jul 28;5:761. doi: 10.1038/s42003-022-03731-z (PMC9334390; doi:10.1038/s42003-022-03731-z)
Supplement: Supplementary file 7 — Reporting Summary [file 42003_2022_3731_MOESM7_ESM.pdf]

nature research

Corresponding author(s): Jianqing Ding, COMMSBIO-21-3382A

Last updated by author(s): Jun 3, 2022

Reporting Summary

Nature Research wishes to improve the reproducibility of the work that we publish. This form provides structure for consistency and transparency in reporting. For further information on Nature Research policies, see our Editorial Policies and the Editorial Policy Checklist.

Statistics

For all statistical analyses, confirm that the following items are present in the figure legend, table legend, main text, or Methods section.

n/a	Confirmed	
☐	☒	The exact sample size (*n*) for each experimental group/condition, given as a discrete number and unit of measurement
☐	☒	A statement on whether measurements were taken from distinct samples or whether the same sample was measured repeatedly
☐	☒	The statistical test(s) used AND whether they are one- or two-sided<br>*Only common tests should be described solely by name; describe more complex techniques in the Methods section.*
☐	☒	A description of all covariates tested
☐	☒	A description of any assumptions or corrections, such as tests of normality and adjustment for multiple comparisons
☐	☒	A full description of the statistical parameters including central tendency (e.g. means) or other basic estimates (e.g. regression coefficient) AND variation (e.g. standard deviation) or associated estimates of uncertainty (e.g. confidence intervals)
☐	☒	For null hypothesis testing, the test statistic (e.g. F, t, r) with confidence intervals, effect sizes, degrees of freedom and P value noted<br>*Give P values as exact values whenever suitable.*
☒	☐	For Bayesian analysis, information on the choice of priors and Markov chain Monte Carlo settings
☐	☒	For hierarchical and complex designs, identification of the appropriate level for tests and full reporting of outcomes
☐	☒	Estimates of effect sizes (e.g. Cohen's d, Pearson's r), indicating how they were calculated

Our web collection on statistics for biologists contains articles on many of the points above.

Software and code

Policy information about availability of computer code

Data collection	No software was used.
Data analysis	SAS for Windows.

For manuscripts utilizing custom algorithms or software that are central to the research but not yet described in published literature, software must be made available to editors and reviewers. We strongly encourage code deposition in a community repository (e.g. GitHub). See the Nature Research guidelines for submitting code & software for further information.

Data

Policy information about availability of data

All manuscripts must include a data availability statement. This statement should provide the following information, where applicable:

- Accession codes, unique identifiers, or web links for publicly available datasets
- A list of figures that have associated raw data
- A description of any restrictions on data availability

The authors declare that the data supporting this study are available within the article’s Supplementary files. Extra data are available from the last author upon request (E.S., siemann@rice.edu).

Field-specific reporting

Please select the one below that is the best fit for your research. If you are not sure, read the appropriate sections before making your selection.

☐ Life sciences ☐ Behavioural & social sciences ☒ Ecological, evolutionary & environmental sciences

For a reference copy of the document with all sections, see nature.com/documents/nr-reporting-summary-flat.pdf

Ecological, evolutionary & environmental sciences study design

All studies must disclose on these points even when the disclosure is negative.

Study description

We conducted a study with wheat aphid and natural enemy data from 120 studies across China and Europe from 1970 to 2017.

We used historical records of temperature (1970-2016), nitrogen fertilizer applications (1980-2015), pesticide applications (1991-2015), and land use (1979-2015) for Chinese provinces and European countries that contributed aphid data, to explore the roles of climate and agricultural practices in determining wheat aphid populations.

Research sample

A total of 2141 data points from China and 1169 data points from Europe in 120 articles were collected for wheat aphids.

A total of 508 data points from China and 123 data points from Europe in 30 articles were collected concerning the population of natural enemies.

We obtained historical records on temperature data for 1970-2016 for the Chinese provinces and European countries that contributed aphid data.

We obtained nitrogen fertilizer application rates for wheat (1980-2015) and pesticide (all types) application rates for all crops combined (1991-2015) from the National Bureau of Statistics of China. We obtained data on nitrogen fertilizer application rates for Western Europe crops (biennial 1980-2010), for cereal crops in the UK (biennial 1984-2014) from the British Survey of Fertiliser Practice and for crops in European countries that contributed aphid data (2002-2015) from FAO. We obtained data on insecticide application rates for all crops combined (1991-2015) for European countries that contributed aphid data from FAO.
We obtained data on areas of land used to grow crops (wheat and all combined) for European countries that contributed aphid data (1979-2015) from FAO. We obtained data on areas of land used to grow crops (wheat and all combined) for Chinese provinces that contributed aphid data (1979-2015) from the China Rural Statistical Yearbooks.

Sampling strategy

We used three databases (Web of Science, Google Scholar and CNKI) to search for studies on populations of wheat aphids between January 1970 and December 2017. We used sets of keywords for study collection to identify the relevant articles: (aphid) and (population OR abundances OR dynamics OR long-term OR time series OR observation) and (wheat). We used the following criteria to screen studies in the dataset: (1) the study was a field survey in open wheat plots, (2) the papers reported aphid data for specific dates within a year, (3) the data of aphid abundances were reported with specific units (per tiller or per m2), (4) the data include all the aphid reported on wheat. We excluded data from treatments or studies that reported insecticide application.

We conducted a literature search (published between January 1970 and December 2017) of natural enemies using the keywords to collect the relevant articles from the three databases used for aphid population collections: (aphid) and (natural enemy* or predator* or parasite*) and (population OR abundances OR dynamics OR long-term OR time series OR observation) and (wheat).

Data collection

We used three databases (Web of Science, Google Scholar and CNKI) to search for studies on populations of wheat aphids between January 1970 and December 2017.

We conducted a literature search (published between January 1970 and December 2017) of natural enemies using the keywords to collect the relevant articles from the three databases used for aphid population collections.

We obtained historical records on temperature data for 1970-2016 for the Chinese provinces and European countries that contributed aphid data.

We obtained data on areas of land used to grow crops (wheat and all combined) for European countries that contributed aphid data (1979-2015) from FAO.

Xiao Sun and Evan Siemann completed the data collection.

Timing and spatial scale

We collected all the wheat aphid, natural enemy, agricultural practices, climate data by website from December 2018 to April 2020.

Data exclusions

No data exclusions.

Reproducibility

Statistical analysis was fully reproduced upon analysis of all data.

Randomization

Statistical analysis was fully randomized upon analysis of all data.

Blinding

All experiments and analyses were performed double blind.

Did the study involve field work? ☐ Yes ☒ No

Reporting for specific materials, systems and methods

We require information from authors about some types of materials, experimental systems and methods used in many studies. Here, indicate whether each material, system or method listed is relevant to your study. If you are not sure if a list item applies to your research, read the appropriate section before selecting a response.

Materials & experimental systems

n/a	Involved in the study
☒	☐ Antibodies
☒	☐ Eukaryotic cell lines
☒	☐ Palaeontology and archaeology
☒	☐ Animals and other organisms
☒	☐ Human research participants
☒	☐ Clinical data
☒	☐ Dual use research of concern

Methods

n/a	Involved in the study
☒	☐ ChIP-seq
☒	☐ Flow cytometry
☒	☐ MRI-based neuroimaging
